# Effect of diindolylmethane supplementation on low-grade cervical cytological abnormalities: double-blind, randomised, controlled trial

**DOI:** 10.1038/bjc.2011.496

**Published:** 2011-11-10

**Authors:** A Castañon, A Tristram, D Mesher, N Powell, H Beer, S Ashman, G Rieck, H Fielder, A Fiander, P Sasieni

**Affiliations:** 1 Centre for Cancer Prevention, Wolfson Institute of Preventive Medicine, Queen Mary University of London, Charterhouse Square, London EC1M 6BQ, UK; 2 Department Obstetrics & Gynaecology, School of Medicine, Cardiff University, Heath Park, Cardiff CF14 4XN, UK; 3 Cervical Screening Wales, 18 Cathedral Road, Cardiff CF11 9LJ, UK

**Keywords:** cervical intraepithelial neoplasia, chemoprevention, diindolylmethane (DIM), randomised, controlled trial, indole-3-carbinol (I3C), human papillomavirus (HPV), food supplement

## Abstract

**Background:**

Cervical screening identifies many women with low-grade abnormalities. *In vitro* and *in vivo* studies have shown that diindolylmethane (DIM) could potentially halt (cervical) carcinogenesis. We report on a randomised controlled trial of the effect of DIM in women with low-grade cervical cytological abnormalities.

**Methods:**

We conducted a pragmatic double-blind, randomised controlled trial of 150 mg DIM (from BioResponse DIM) or placebo daily for 6 months in women with newly diagnosed, low-grade cytological abnormalities. Randomisation was in the ratio 2 (DIM) to 1 (placebo). All women were invited for colposcopy at 6 months with biopsy of any abnormality.

**Results:**

Of the 551 randomised women available for analysis, 9% on DIM and 12% on placebo had cervical intraepithelial neoplasia-2 (CIN2) or worse after 6-month supplementation (risk ratio (RR) 0.7 (95% confidence interval (CI): 0.4–1.2)), whereas 4.6% and 5.1%, respectively, had CIN3 or worse (RR 0.9 (95% CI: 0.4–2.0)). A total of 27.3% of women on DIM and 34.3% on placebo had no sign of disease (negative cytology, colposcopy and human papilloma virus (HPV) tests) at 6 months (RR 0.8 (95% CI: 0.6–1.0)). Of those HPV-positive at baseline, 69% (114 out of 166) of the DIM group were positive at 6 months compared with 61% (43 out of 71) of the placebo group: RR 1.1 (95% CI: 0.9–1.4). Diindolylmethane supplementation was well tolerated.

**Conclusion:**

The results suggest that short-term DIM supplementation (150 mg day^−1^) is well tolerated, but is unlikely to have an effect on cytology or HPV infection. Uncertainty remains regarding its effect on CIN2+.

In the United Kingdom, approximately 6% of cervical screening cytology is classified as a low-grade abnormality (borderline changes or mild dyskaryosis). At the time of this study, the management of women with low-grade cytological abnormalities was covered by the Cervical Screening Wales: National Service Framework for the Cervical Screening Programme in Wales, 2009. After one such abnormal result, women are advised to have a repeat cytology every 6 months until they have three consecutive negative results. If a second (in the case of mild dyskaryosis) or third test (in the case of a borderline abnormality) is abnormal, they are referred to colposcopy for further assessment (including biopsy if indicated) and treatment as necessary. If a low-grade lesion is confirmed on histology, management may be conservative, but if a high-grade lesion is identified excisional, treatment is recommended. Both women and the health service would benefit from the existence of an effective natural medical treatment (using an oral preparation) for low-grade cytological lesions that would increase regression and reduce the need for excision.

Indole-3-carbinol (I3C) and its dimer diindolylmethane (DIM) are found in cruciferous vegetables (Ciska *et al*, 2009) and have been identified as compounds that could potentially prevent or halt carcinogenesis (Bradlow, 2008; Ahmad *et al*, 2010). Diindolylmethane is naturally formed from I3C during acid digestion of cruciferous vegetables and I3C supplements (Reed *et al*, 2006; Ciska *et al*, 2009). It has been shown that DIM acts directly to cause apoptosis in human breast, endometrial, cervical, ovarian, prostate, and colon tumour cells in culture (Ge *et al*, 1996; Bonnesen *et al*, 2001; Chen *et al*, 2001; Hong *et al*, 2002; Nachshon-Kedmi *et al*, 2004; Azmi *et al*, 2008). When BioResponse DIM was administered orally to human papilloma virus-16 (HPV-16) transgenic mice, it was found to inhibit cervical dysplasia, alter oestrogen metabolism, and enhance immune response (Sepkovic *et al*, 2009).

The BioResponse formulation of DIM has been allowed unrestricted human use as a dietary supplement in the United States of America under the Dietary Supplement Health and Education Act of 1994. The known side effects, nausea, dyspepsia, and flatulence, are mild, reversible, and uncommon. There have been reports that DIM may increase the severity of headaches in women who get migraine. Many subjects also report a harmless darkening of urine owing to excretion of coloured DIM metabolites. A previous controlled trial reported that DIM relieved recurrent breast pain (cyclical mastalgia) (Zeligs *et al*, 2005). Additionally DIM seems to result in an increase in afternoon ketosis and may facilitate weight loss in women on a low-carbohydrate diet (Zeligs, 2003).

The objectives of this study were to assess, in a routine screening programme setting, the effect of 6-month supplementation with DIM on (i) histologically proven high-grade cervical intraepithelial neoplasia (CIN); (ii) the prevalence of cytological abnormalities; (iii) cervical HPV infections; and (iv) to assess any side effects of supplementation. A pre-planned sub-study monitored the effects of DIM on weight change, headache, mastalgia, and premenstrual syndrome.

## Materials and methods

### Study population

Women were eligible for this study if they were advised to have a repeat screen at 6 months after a first mildly dyskaryotic smear or a second borderline smear taken within the quality-assured Cervical Screening Wales (CSW) programme. Recruitment to the trial was performed between October 2004 and December 2007. Women identified through the CSW programme as eligible were approached using a leaflet sent through post. Interested women were asked to contact the research team to set up an appointment and check eligibility for the study. Women were not eligible for the study if they were known to be pregnant, breastfeeding, HIV-positive, taking immunosuppressive drugs or proton pump inhibitors, under surveillance having been previously treated for high-grade CIN, diagnosed with invasive cancer in the previous 3 years, or if there was a clinical suspicion of invasive cervical cancer. Women were excluded from analysis if they were treated for CIN 0–4 months after entry into the study.

The study was a double-blind, randomised, controlled, primary prevention trial where participants were asked to take either 150 mg DIM from BioResponse DIM (BioResponse, LLC, Boulder, CO, USA) or a placebo (provided as identical oral capsules containing calcium carbonate, maltodextrin (a complex carbohydrate sourced from corn starch), and the colouring agent Opadry NS Orange) every day for 6 months. Participants were in the ratio 2 : 1 for DIM : placebo. Randomisation was in blocks of random size 6 or 9 to ensure balance using an in-house computer programme. Once entered into the study, patients were randomly allocated a patient number that determined their supplement allocation, and four bottles of capsules pre-labelled with their study number were dispensed. The four bottles contained sufficient capsules for 6 months.

Women in the trial were offered up to three appointments for colposcopy before being re-incorporated into the CSW programme for further follow-up. Active participation in the study ended on 12 May 2008 after the last enrolled participant attended her 6-month appointment. Histological or cytological results taken outside of the trial were identified using the CSW database (up until March 2009).

The South East Wales Local Research Ethics Committee approved the research and informed written consent was obtained from each participant before randomisation. An independent Data and Safety Monitoring Committee was in place throughout the trial to review study progress, potential side effects, and efficacy. The trial is registered at ClinicalTrials.gov (ClinicalTrials.gov, number NCT00462813) and ISRCTN (ISRCTN Register, number 47437431).

### Data collection

At the initial consultation a baseline questionnaire ascertained information on reproductive history, diet, smoking, current medication, history of migraine, and premenstrual symptoms. Additionally women were weighed and a cervical sample was obtained for HPV testing. Women received four bottles, pre-labelled with their study number, containing sufficient capsules of either BioResponse DIM or placebo for 6 months. The appearances of the bottles and capsules were identical in the two arms. All clinic personnel and study participants were blinded to treatment assignment for the duration of the study.

Three months after entry participants were invited to attend a nurse-led clinic at which a questionnaire was administered regarding compliance and side effects. Women who did not attend this visit were sent the questionnaire through post.

Six months after entry into the study, all women were offered colposcopy. At the colposcopy visit women were weighed, any unused were capsules retrieved, a smear was taken for liquid-based cytology, colposcopy was performed, any visible lesions were biopsied, and a questionnaire was completed.

### Endpoint ascertainment

Histology and cytology results were cross-checked with the CSW Programme database and any discrepancies were verified with the original data source. Using this database we also identified further histology or cytology results (up until March 2009) taken outside of the trial. The intention was to study the effects of 6 months of supplementation on cervical pathology; therefore cytology results 0–4 months after randomisation were excluded. The cytology results presented here are from the study visit if that visit was between 4 and 8 months after randomisation. If no such study result is available then the earliest result (including both study and routine tests) 4–12 months after entry is used. Histology and colposcopy results were included if they were taken 0–3 months after the selected cytology result.

The primary endpoint for this study was presence of disease defined as histologically confirmed CIN2+. Other endpoints were high-risk human papillomavirus (HPV) positivity on a sample taken at the 6-month visit, stratified by initial HPV result; the presence of CIN3+ and post-treatment cytology result.

Smears and histological samples were read and reported within the routine CSW programme. Cervical cytology was reported as normal, borderline nuclear changes, mild dyskaryosis, moderate dyskaryosis, severe dyskaryosis, possible invasion, and glandular neoplasia. As specified in the statistical analysis plan, cytology was analysed in three ordered categories, normal, low-grade (borderline or mild), and high-grade (moderate, severe, or worse), corresponding to the most common groupings for clinical management. HPV testing was performed at the HPV laboratory, University Hospital of Wales, by PCR enzyme immunoassay (PCR-EIA) using GP5+/6+ primers (Jacobs *et al*, 1997). Sample adequacy was determined by inclusion of a *β*-globin probe. Those samples identified as containing high-risk HPV types were then subjected to a second PCR-EIA using individual oligonucleotide probes to obtain genotype data. Samples were considered HPV-positive if they were positive for a high-risk HPV both using the consensus primers and on the type-specific assay.

### Sample size

We anticipated that the incidence of CIN2 or CIN3 at 6 months would be 7.2% in the DIM group and 12% in those taking placebo. Based on 90% power to detect a significant difference (*P*=0.05, two-sided) in histology, 3000 participants were required. We had (naively) assumed that we could recruit 3000 women over 3 years (with 50% of eligible women participating). In practice only 11% of eligible women were randomised as 83% did not respond to the initial mailed letter inviting them into the study. The trial failed to recruit the desired number of women, and owing to lack of further funds recruitment ended when 603 women had been enrolled. Early stopping after recruitment of 600 patients was discussed in the protocol and provided over 90% power for the secondary endpoints: negative cytology at 6 months and HPV regression.

### Statistical analysis

The main statistical analyses were performed on all randomised women for whom outcome data were available.

The primary outcome was histologically confirmed CIN2+ *vs* histology without CIN2+ or negative colposcopy (and no histology).

Comparison of binary outcomes between treatment groups was made by 2 × 2 tabulation and a risk ratio (RR), 95% confidence intervals (CIs), and *P*-values were calculated. The significance of trends in ordered categorical outcomes between the two treatment groups specified in the statistical analysis plan was assessed using the normal approximation of the Wilcoxon Rank Sum test (with an appropriate correction for ties using the ranksum command in STATA).

Adverse events (including serious adverse events) were tabulated by treatment group, and RR, 95% CIs, and *P*-values were calculated. Any adverse events (not considered to be serious) previously associated with DIM, which occurred in 5% or more women in either group, which had a 2% or more difference in prevalence between treatment groups or which were significantly different at the 5% significance level, were included. Events that were non-serious, unexpected, and rare are not presented.

Analyses were performed using STATA 10 (StataCorp. 2007. *Stata Statistical Software: Release 10*; College Station, TX, USA: StataCorp LP).

## Results

**Figure 1:**
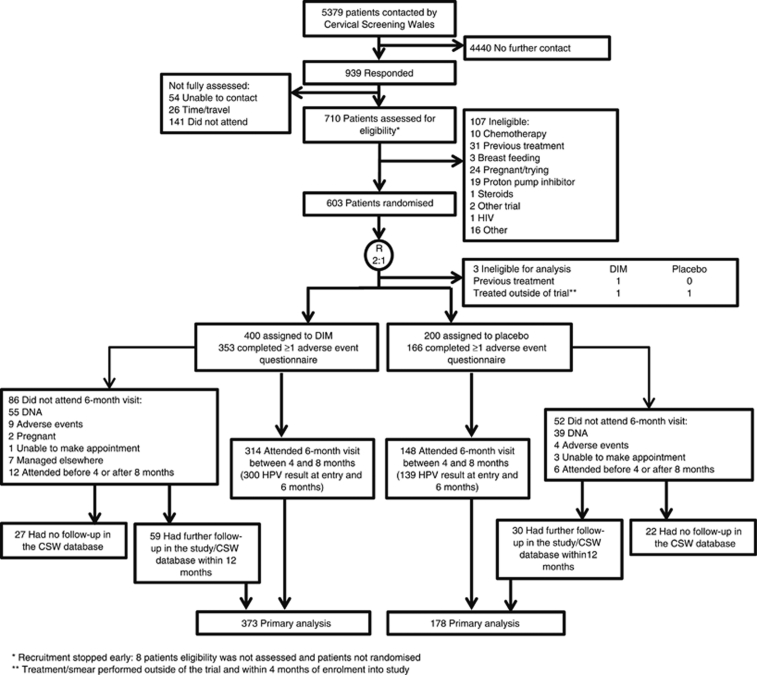
Patient enrolment and compliance.

A total of 710 women were assessed for eligibility of which 603 were randomised. The reasons for exclusion are detailed in Figure 1. Out of the 603 women randomised, three were considered ineligible for analysis (1 had been previously treated and 2 had treatment within 4 months of enrolment into the study). Of the remaining women 400 were assigned to DIM and 200 to placebo. Of the 480 women who attended the study colposcopy, the results of 18 are excluded because the visit was not between 4 and 8 months of entry. Of the remaining 120 women who did not attend their 6-month study visit, 94 gave no reason, 13 withdrew owing to adverse events, 2 became pregnant, 4 were unable to make an appointment with the clinic, and 7 were managed elsewhere. We were able to obtain cytology/histology results using further cytology within 12 months available from the study or through the CSW database for 89 of the 138 (64%) women who did not attend their 6-month visit. The intention-to-treat analysis was based on 373 women in the DIM group and 178 women in the placebo group (Figure 1). A total of 439 women had adequate HPV samples at both entry and 6 months. These numbers provided 30.3% power for the primary endpoint (CIN2+), 95.6% power for the cytology endpoint, and 63.4% power for the clearance of HPV endpoint.

There were three protocol violations. One woman had been treated for CIN2 prior to recruitment. This woman was withdrawn from the study within a month of randomisation as described above. Another took a proton pump inhibitor for approximately 3 months while in the study; the trial nurses only realised this once the woman had completed the trial. The third received colposcopy, without treatment, at her 3-month visit (it was repeated at her 6-month visit). Neither of these two women were excluded from the study.

Baseline characteristics did not differ between the two arms (Table 1). Mean age was 36 years (range 19–65). Mean weight was 69 kg, with 38% of women over 70 kg at baseline. Approximately one-third of women were on hormonal contraceptives during the study and only 20% reported eating more than five portions a week of cruciferous vegetables such as cabbage, cauliflower, Brussels sprouts, or broccoli. Just over a quarter of women were current smokers, but very few of these smoked over 20 cigarettes a day. Approximately half the participants were enrolled with borderline cytology and the other half with mild dyskaryosis.

A non-significantly higher proportion of women on DIM attended their 6-month (78.5%) visit than those on placebo (74%) (*P*=0.217). The proportion of women who did not attend their 6-month appointment but for whom further follow-up cytology was identified in the study or on the CSW database was similar in both groups (69% for DIM and 58% for placebo). Compliance was self-reported on the 3- and 6-month questionnaires, and in addition unused capsules were counted when returned at the 6-month visit. There were no significant differences in compliance between the groups (data not shown).

The primary and secondary outcome results for women included in the analysis are presented in Table 2. Of the 551 women available for analysis, 8.8% on DIM and 12.4% on placebo were diagnosed by histology with CIN2 or worse (RR 0.70 (95% CI: 0.4–1.2)). Stratified analysis of the relative risk of CIN2 or worse by baseline characteristics is shown in Supplementary Figure S1. For all baseline characteristics, the difference in DIM efficacy (as measured by relative risk) between strata was non-significant (*P*>0.05). The smallest relative risk (greatest DIM efficacy) was observed in ex-smokers (RR 0.33 (95% CI: 0.11–0.97)). However, both ex-smokers and never smokers had relative risk greater than the overall relative risk (i.e., >0.70).

Of the women on DIM, 4.6% were diagnosed with CIN3 or worse compared with 5.1% on placebo (RR 0.9 (95% CI: 0.4–2.0)), whereas 50.0% and 55.7%, respectively, had negative cytology at the end of the study (RR 1.13 (95% CI: 0.93–1.37)). 27% of women on DIM and 34% of women on placebo had negative cytology, colposcopy, and HPV tests at 6 months (RR 0.8 (95% CI: 0.6–1.0)).

Ninety-four percent (439 out of 462) of those women who attended the 6-month appointment had an adequate HPV test at entry and at 6 months. Of those positive at baseline, 69% (114 out of 166) of the DIM group were positive at 6 months compared with 61% (43 out of 71) of the placebo group (RR 1.1 (95% CI: 0.9–1.4)) (Table 2).

Of those who were HPV-negative at baseline, 7% (10 out of 134) on DIM and 9% (6 out of 68) on placebo became HPV-positive by 6 months (RR 0.8 (95% CI: 0.3, 2.2)). Analysis of HPV status at 6 months stratified by baseline HPV status gives a combined RR of 1.1 (95% CI: 0.9, 1.4).

More detailed cytology and histology results are presented in Table 3. A larger proportion of women on DIM had a borderline or worse cytology at follow-up compared with placebo (50% *vs* 44%, respectively). However both groups had a similar proportion with histologically confirmed CIN1 or worse (22% of women on DIM and 20% on placebo).

There was no difference in the proportion of women in both groups with persistent HPV types at 6 months. Women in the DIM group were slightly and non-significantly less likely to develop an incident infection with HPV-16 (2.8%) or HPV-18 (2.0%) than women on placebo (3.7% and 4.3%, respectively) (Table 4).

### Adverse events

A total of 10 serious adverse events were reported: 4 (1.0%) occurred in the DIM group and 6 (3.0%) occurred in the placebo group (RR 0.3 (95% CI: 0.1–1.2)); none were life-threatening. Three were reported as suspected unexpected serious adverse reactions (SUSARs): (1) A spontaneous abortion (on placebo). (2) A woman (on DIM) with previous fibroids was admitted to hospital because of abdominal pain: her fibroids had increased in size since her last ultrasound some time previous to recruitment into the trial. (3) A woman (on DIM) with a history of intermittent inter-menstrual bleeding, which became worse during the study and was treated by thermal ablation. Adverse events reported during the study are presented in Table 5. Non-serious adverse events were reported for women responding to at least one of the 3- or 6-month questionnaires on adverse events (*n*=353 in the DIM arm and *n*=166 in the placebo arm). The most common known side effect of DIM was darkening of urine, which was reported by 31% of women on DIM compared with 10% of women on placebo (*P*<0.0001). Not including darkening of urine, 69% of women on DIM reported (one or more) non-serious adverse events compared with 60% on placebo.

Self-reported weight gain was rare but statistically significantly different between the DIM and placebo groups: 0.3% of women on DIM compared with 3.0% of women on placebo (*P*=0.03). We examined changes in weight as measured at baseline and 6 months and found no evidence of differences in weight change between the two treatment groups (*P*=0.62). There was a non-significant differential improvement in self-reported premenstrual syndrome (PMS), with 50% (68 out of 135) of women in the DIM arm who had PMS at baseline and reported on it subsequently having some improvement compared with 40% (24 out of 60) on placebo (*P*=0.24). There were too few women (*n*=14) with mastalgia to study the effect of DIM on breast pain in this trial.

## Discussion

Six-month supplementation with DIM did not increase the rate of HPV clearance during the period of supplementation in women with low-grade cytological abnormalities, nor did it increase the proportion with negative cytology. This trial was too small to provide narrow CIs on the effect of oral DIM on histology in women with low-grade cytological abnormalities. Although the results from this trial cannot exclude the possibility that 6-month supplementation with DIM could reduce the risk of developing CIN2 or worse by 50% (the 95% CI for the RR included 0.5), it is unlikely that short-term DIM supplementation has a clinically important effect on cytology or HPV persistence. Based on these two secondary endpoints, it seems unlikely that DIM supplementation has a substantial effect on the risk of developing CIN2 in women with low-grade cytology.

The main limitation of this trial is the lack of power (only 30.3%) to address the primary endpoint owing to an insufficient number of women being recruited. That said, with 603 patients randomised, this is still (as far as we are aware) the largest chemoprevention trial in women with either cytological or histological cervical abnormalities; and the power to detect a significant difference in the proportions with normal cytology at 6 months was 95.6%. It was disappointing that fewer than 20% of those informed about the study responded, but we only had permission from Research Ethics to approach women by post. This trial intended to fit in with the cervical screening programme in the United Kingdom so it was essential to recruit women very shortly after their abnormal screen so as to be able to accommodate 6-month supplementation without delaying their early recall. Future studies should consider a personal approach to potentially eligible patients by their nurse or doctor, rather than an impersonal letter as was used in this trial. The baseline characteristics between groups suggests that randomisation for this trial was adequate, and although 20% of women did not attend their 6-month appointment, we were able to trace cytology, colposcopy, and histology results for half of these women. There is no evidence that compliance was different between groups. Thus, although uptake was poor, the randomisation and blinding provide validity for our finding that 6-monthly supplementation with 150 mg BioResponse DIM once daily is not effective in treating early cervical lesions or HPV infection. A second limitation is that the once-daily administration maybe sub-optimal as the typical plasma half-life for DIM from BioResponse DIM is about 3 h (Reed *et al*, 2008; Heath *et al*, 2010); while drug kinetics may not predict pharmacokinetics, these results suggest the need for studies of multiple daily doses to increase tissue exposure.

It is likely that the women randomised were heterogeneous regarding their underlying histology. This was a pragmatic trial targeted at a group of patients who would be routinely put on 6-monthly surveillance to see whether their cytological abnormality persisted or regressed. We chose not to subject women to a baseline biopsy because we wanted to continue ‘standard management’ and take advantage of the 6-month window waiting for repeat cytology. Further, a biopsy may affect the natural history of the lesion, and had we found CIN2 or CIN3 we would have been obliged to treat the patient and exclude her from the trial.

Other trials have studied I3C and DIM in cervical neoplasia. In a small randomised, controlled clinical trial using I3C for 12 weeks, in women with biopsy-confirmed high-grade CIN (HG CIN), none of 10 placebo-controlled patients had complete regression of CIN, compared with 4 of 8 patients randomised to receive 200 mg day^−1^ I3C and 4 of 9 in the 400-mg day^−1^ arm (Bell *et al*, 2000). A second trial recruited 64 women with biopsy-proven CIN2 or CIN3 randomised 2 : 1 to BioResponse DIM (2 mg kg^−1^  per day) or placebo for 3 months (Del Priore *et al*, 2010). After between 12 weeks and 12 months of follow-up, approximately two-third of the lesions had regressed (to CIN1 or less) in both groups. There was no evidence of a therapeutic effect in terms of histology, cytology, or colposcopy outcome. It has been suggested that the protective effects of indoles may be influenced by individual genetic variation (polymorphisms) in the metabolism and elimination of isothiocyanates from the body (Higdon *et al*, 2007). The outcome of the very small trial of I3C is inconsistent with our own findings and of the smaller US trial using DIM. Although it is possible that the different outcomes are due to the difference between I3C and DIM, it seems likely to us that the dramatic results of the very small trial of I3C were a chance finding.

There are now considerable safety data on DIM from BioResponse DIM in humans (Zeligs *et al*, 2005; Reed *et al*, 2008; Del Priore *et al*, 2010; Heath *et al*, 2010). In the study reported here, there was no statistically significant difference in serious adverse events between groups; in fact a higher proportion of women in the placebo group reported a serious adverse event. The most common side effect of DIM was darkening of urine, which is due to a harmless by-product. There are anecdotal reports that DIM may increase the severity of migraine headaches. Although this study did not have sufficient power to study migraines, we did find a non-significant increase in reported headaches (18% on DIM, 12% on placebo, *P*=0.12). Even excluding darkening of urine, women on DIM were more likely than women on placebo to experience an adverse event, but no single adverse event, other than darkening of urine, was reported significantly more often in the DIM group than the placebo group.

This trial was conducted in women with borderline changes or mildly dyskaryosis on cervical cytology in a screening programme that managed such women by 6-month repeat cytology. Just over 50% of those recruited tested positive for HPV at entry. Where primary cervical screening is by HPV testing, it would be valuable to be able to offer a treatment such as DIM supplementation to women screening positive for HPV but who are negative on a triage test. This trial does not directly address that issue, but does not provide any encouragement in terms of the efficacy of DIM supplementation in such a population (the proportion with HPV clearance was non-significantly increased in the DIM arm of this study). There is also interest in using DIM in women with vulva intraepithelial neoplasia. Any such study would need to address the issue of the 3-hour half-life of BioResponse DIM. There remains a general need for safe medical treatment of intraepithelial neoplasia and HPV infection. This randomised, double-blind trial suggests that 150 mg DIM once daily is not effective in promoting HPV clearance or in preventing CIN progression in women with mildly abnormal cervical cytology.

## Figures and Tables

**Table 1:** Baseline data and demographics^a^

	**DIM (*n*=400)**	**Placebo (*n*=200)**
Age^b^ (years)	36.7±11.7 (19, 65)	35.8±12.1 (20, 65)
Weight^c^ (kg)	68.8±14.3	69.4±14.1
		
*Contraceptive use*		
Hormonal only	142 (35.5%)	76 (38.0%)
Barrier only	56 (14.0%)	28 (14.0%)
Other	34 (8.5%)	21 (10.5%)
Sterile	63 (15.8%)	22 (11.0%)
Contraception not required	99 (24.8%)	52 (26.0%)
		
*Portions of vegetables^d^ per week*		
None	51 (12.8%)	38 (19.0%)
1–2	150 (37.5%)	59 (29.5%)
3–4	108 (27.0%)	62 (31.0%)
5+	82 (20.5%)	38 (19.0%)
		
*Smoking status*		
Never	240 (60.0%)	124 (62.0%)
Ex	47 (11.8%)	17 (8.5%)
<10 per day	54 (13.5%)	26 (13.0%)
10–19 per day	43 (10.8%)	26 (13.0%)
20+ per day	10 (2.5%)	6 (3.0%)
		
*Migraines*		
No	299 (74.8%)	151 (75.5%)
Yes	99 (24.8%)	47 (23.5%)
		
*Premenstrual syndrome*		
No	220 (55.0%)	107 (53.5%)
Yes	164 (41.0%)	84 (42.0%)
		
*Mastalgia*		
No	238 (59.5%)	125 (62.5%)
Yes	147 (36.8%)	65 (32.5%)
		
*Cytology*		
Mild	198 (49.5%)	95 (47.5%)
Borderline	202 (50.5%)	105 (52.5%)

Abbreviation: DIM=diindolylmethane.

a Values are means±s.d. (range) or number (%) as appropriate.

b No women were missing age.

c 22 (11.0%) and 30 (7.5%) women missing weight for placebo and DIM, respectively.

d Vegetables include cabbage, broccoli, or similar.

**Table 2:** Primary and secondary outcomes at follow-up (including 6 months) for women with 6-month visit^a^

	**DIM** a	**Placebo** a	**Relative risk (95% CI)**	***P*-value**
*All women*	*n*=373	*n*=178		
				
*Primary outcome*				
Cervical intraepithelial neoplasia grade 2+	33 (8.8%)	22 (12.4%)	0.7 (0.4, 1.2)	0.198
				
*Secondary outcomes*				
Cytology				
Negative	185 (50.0%)	98 (55.7%)		
Low-grade	159 (43.0%)	66 (37.5%)		0.214^b^
High-grade	26 (7.0%)	12 (6.8%)		
Cervical intraepithelial neoplasia grade 3+	17 (4.6%)	9 (5.1%)	0.9 (0.4, 2.0)	0.796
All negative (Cyt, Colp and HPV)	102 (27.3%)	61 (34.3%)	0.8 (0.6, 1.0)	0.092
				
*Human papillomavirus (HPV)-positive at baseline* c	*n*=166	*n*=71		
*Secondary outcomes*				
HPV-positive at follow-up	114 (68.7%)	43 (60.6%)	1.1 (0.9, 1.4)	0.25
				
*Human papillomavirus (HPV)-negative at baseline* d	*n*=134	*n*=68		
*Secondary outcomes*				
HPV-positive at follow-up	10 (7.5%)	6 (8.8%)	0.8 (0.3, 2.2)	0.735
Combined HPV-positive at follow-up	124 (41.3%)	49 (35.3%)	1.1 (0.9, 1.4)	0.386

Abbreviations: CI=confidence interval; DIM=diindolylmethane.

a Values are number (%) as appropriate.

b *P*-value compares negative with non-negative cytology.

c Numbers are those with non-missing HPV result at 6 months; 3 missing 6-month HPV result in placebo and 3 missing 6-month HPV result in DIM.

d Numbers are those with non-missing HPV result at 6 months; 1 missing 6-month HPV result in placebo and 8 missing 6-month HPV result in DIM.

**Table 3:** Cytology, HPV, and histology results according to treatment group for women with 6-month visit^a^

	**DIM (*n*=373)**	**Placebo (*n*=178)**
*Cytology*		
Inadequate	1 (0.3%)	1 (0.6%)
Normal	185 (49.9%)	98 (55.4%)
Borderline	87 (23.5%)	40 (22.6%)
Mild	72 (19.4%)	26 (14.7%)
Moderate	19 (5.1%)	6 (3.4%)
Severe	7 (1.9%)	6 (3.4%)
Invasive carcinoma/glandular neoplasm	0 (0.0%)	0 (0.0%)
Missing	2	1
		
*Human papillomavirus (HPV)*		
Negative	94 (63.5%)	179 (59.1%)
Positive	50 (33.8%)	124 (40.9%)
Missing	4	11
HPV-16-positive	18 (12.2%)	51 (16.8%)
HPV-16 or HPV-18-positive	26 (17.6%)	60 (19.8%)
		
*Colposcopy/histology*		
Missing colposcopy/no biopsy	42	20
Negative colposcopy^b^	149 (45.0%)	66 (41.8%)
Adequate biopsy	172 (52.0%)	86 (54.4%)
Negative	57 (17.2%)	33 (20.9%)
HPV/borderline	33 (10.0%)	18 (11.4%)
CIN1	49 (14.8%)	13 (8.2%)
CIN2	16 (4.8%)	13 (8.2%)
CIN3	17 (5.1%)	9 (5.7%)
Adeno *in situ*	0 (0.0%)	0 (0.0%)
Missing biopsy^c^	10 (3.0%)	6 (3.8%)

Abbreviations: CIN=cervical intraepithelial neoplasia; DIM=diindolylmethane; HPV=human papilloma virus.

a Values are number (%) as appropriate.

b Negative or HPV-alone colposcopy, and negative, borderline, or mild cytology.

c No or inadequate biopsy with ⩾low-grade colposcopy or ⩾moderate cytology.

**Table 4:** HPV typing results among those with a 6-month visit

		**Type-specific HPV-positive at baseline**			**Type-specific HPV-negative at baseline**		**Stratified RR of type-specific positive at 6 months**
		**Placebo**	**DIM**		**Placebo**	**DIM**	**OR (95% CI)**
High-risk 16	N	20	72	N	128	242	0.9 (0.6, 1.3)
	Persistent	13 (65.0%)	44 (61.1%)	Gain	5 (3.9%)	7 (2.9%)	
High-risk 18	N	13	26	N	135	288	0.7 (0.4, 1.3)
	Persistent	6 (46.2%)	12 (46.2%)	Gain	6 (4.4%)	5 (1.7%)	
Other high-risk	N	65	126	N	83	188	1.1 (0.9, 1.5)
	Persistent	37 (56.9%)	82 (65.1%)	Gain	6 (7.2%)	16 (8.5%)	

Abbreviations: CI=confidence interval; DIM=diindolylmethane; HPV=human papilloma virus; OR=odds ratio.

**Table 5:** Adverse events (including deaths and serious adverse events) and secondary findings

	**DIM** a	**Placebo** a	**RR (95% CI)**	***P*-value** b
All women	*n*=400	*n*=200		
Any serious adverse Event	4 (1.0%)	6 (3.0%)	0.3 (0.1, 1.2)	0.15
Death	0 (0.0%)	0 (0.0%)	—	—
Women completing questionnaire	*n*=353	*n*=166		
Darkening of urine	110 (31.2%)	16 (9.6%)	3.2 (2.0, 5.3)	<0.0001
Any non-serious adverse event^c^	243 (68.8%)	99 (59.6%)	1.2 (1.0, 1.3)	0.011
				
*Pre-defined adverse events* d				
*Change in bowel frequency*				
Increased	87 (24.6%)	31 (18.7%)	1.3 (0.9, 1.9)	0.159
Decreased	2 (0.6%)	0 (0.0%)	—	0.888
Diarrhoea	13 (3.7%)	4 (2.4%)	1.5 (0.5, 4.6)	0.638
Flatulence	63 (17.8%)	38 (22.9%)	0.8 (0.5, 1.1)	0.218
Headaches	63 (17.8%)	20 (12.0%)	1.5 (0.9, 2.4)	0.116
Nausea	24 (6.8%)	7 (4.2%)	1.6 (0.7, 3.7)	0.338
Skin rash	16 (4.5%)	7 (4.2%)	1.1 (0.5, 2.6)	1
Vomiting	7 (2.0%)	1 (0.6%)	3.3 (0.4, 26.5)	0.432
				
*Other adverse events* e				
Changes to the menstrual cycle	44 (12.5%)	13 (7.8%)	1.6 (0.9, 2.9)	0.149
Self-reported increase in weight	1 (0.3%)	5 (3.0%)	0.1 (0.0, 0.8)	0.028
				
*Secondary findings*				
*Premenstrual syndrome*				
Improved^f^	68/135	24/60	1.3 (0.9, 1.8)	0.236
Weight change^g^	*n*=243	*n*=110		
Mean (kg)	0.22	0.41		0.624
Loss of ⩾2 kg	35/243	13/110	1.2 (0.7, 2.2)	0.634

Abbreviations: CI=confidence interval; DIM=diindolylmethane; RR=risk ratio.

a Values are number (%).

b *P*-values 2^*^ 1-sided Fisher's exact test.

c Any non-serious adverse events excluding darkening of urine.

d The total is greater than the number of women with any event owing to women with multiple types of adverse events.

e Other adverse events are those, which are common in either group or show significant difference as described under Materials and methods.

f Self-reported improvement at either 3 or 6 months (provided not worse on other visit) in those with PMS at baseline who reported on PMS at either 3 or 6 months.

g In those with a 6-month study visit between 4 and 7 months, and a valid weight at entry and 6-month visit.
