# Supplementary material for: Effect of diindolylmethane supplementation on low-grade cervical cytological abnormalities: double-blind, randomised, controlled trial
Source: Br J Cancer. 2011 Nov 10;106(1):45–52. doi: 10.1038/bjc.2011.496 (PMC3251847; doi:10.1038/bjc.2011.496)
Supplement: Supplementary Figure S1 [file bjc2011496x1.ppt]

## Slide 1
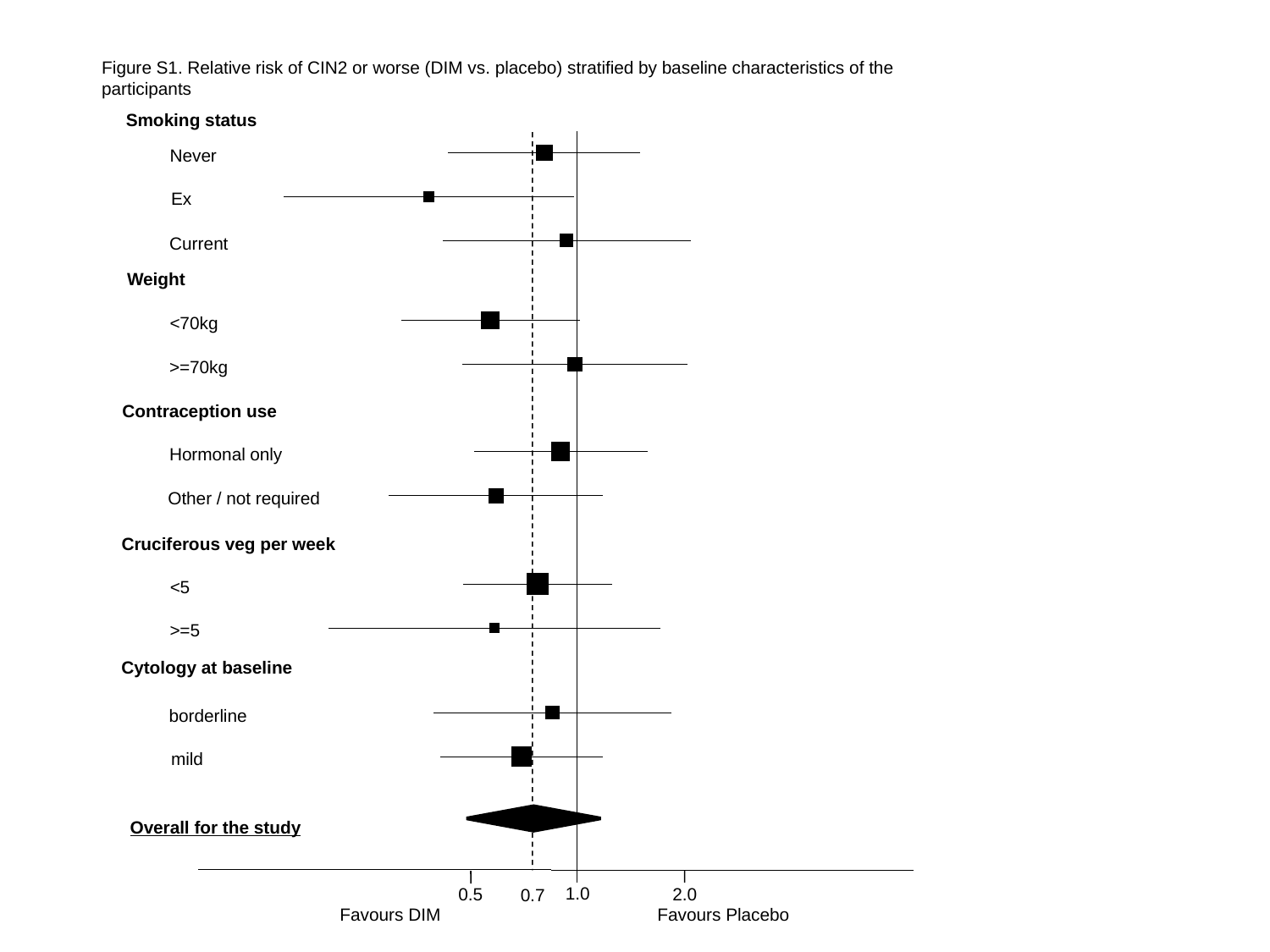

Figure S1. Relative risk of CIN2 or worse (DIM vs. placebo) stratified by baseline characteristics of the participants
Smoking status
Never
Ex
Current
Weight
<70kg
>=70kg
Contraception use
Hormonal only
Other / not required
Cruciferous veg per week
<5
>=5
Cytology at baseline
borderline
mild
Overall for the study
Favours DIM
Favours Placebo
1.0
0.5
2.0
0.7
